# Covert Timing Channel Analysis Either as Cyber Attacks or Confidential Applications

**DOI:** 10.3390/s20082417

**Published:** 2020-04-24

**Authors:** Shorouq Al-Eidi, Omar Darwish, Yuanzhu Chen

**Affiliations:** 1 Computer Science Department, Memorial University of Newfoundland, St. John’s, NL A1B 3R7, Canada; yzchen@mun.ca; 2 Computer Information System Department, Ferrum College, Ferrum, VA 24088, USA; odarwish@ferrum.edu

**Keywords:** information security, computer networks, covert timing channels, inter-arrival times, Internet of Things

## Abstract

Covert timing channels are an important alternative for transmitting information in the world of the Internet of Things (IoT). In covert timing channels data are encoded in inter-arrival times between consecutive packets based on modifying the transmission time of legitimate traffic. Typically, the modification of time takes place by delaying the transmitted packets on the sender side. A key aspect in covert timing channels is to find the threshold of packet delay that can accurately distinguish covert traffic from legitimate traffic. Based on that we can assess the level of dangerous of security threats or the quality of transferred sensitive information secretly. In this paper, we study the inter-arrival time behavior of covert timing channels in two different network configurations based on statistical metrics, in addition we investigate the packet delaying threshold value. Our experiments show that the threshold is approximately equal to or greater than double the mean of legitimate inter-arrival times. In this case covert timing channels become detectable as strong anomalies.

## 1. Introduction

Covert channels are communications channels used to transmit information using existing system resources that were not designed to carry data without being detected by network security mechanisms, such as firewalls. Because of their ability to evade detection, they are create a grave cyber security threat. Sensitive and confidential information can be leaked from an entity with higher security privileges to another entity by establishing a seemingly innocuous communication channel between them. Two parties with the intention to transmit covert information can easily communicate and exchange information over a shared network without being detected. Therefore, it is difficult to detect covert communications, and this can be effective and damaging security approaches if it is used for malicious intentions. Depending on the type of methods used for hiding information, there are two main categories of covert channels: covert storage channels (CSCs) and covert timing channels (CTCs). In CSCs, the sender directly writes covert information into network objects, such as protocol header fields and extensions, while the receiver can read covert information from the same network objects [1]. On the other hand, in CTCs, the sender embeds covert information by modulating the time information of legitimate traffic. The receiver observes the covert traffic, extracts the timing information from it, and decodes the covert information [2]. CSCs are bounded by network protocols, and hence they can not deviate from certain behavior. This makes detecting them not difficult. On the other hand, CTCs show stochastic behaviour and hence detecting them is more challenging.

While most of the existing CTCs are usually used for malicious intentions such as malware spreading and industrial espionage, these channels can be used as alternative techniques for transmitting sensitive information in untrusted networks. For example, due to the lack of central control and fixed infrastructure, limited energy resources, the burden of the flexibility of information processing, and network access are put on smart things, which refer to a wide variety of smart devices in the Internet of Things (IoT), the privacy of transmission the confidential information in an untrusted IoT has become a huge obstacle to the popularity of technology [3]. For more specific, sending sensitive information using 5G or 6G networks in different IoT applications, including the Industrial IoT, cloud services, edge computing, network virtualization, healthcare devices, and authentication devices, will bring new security vulnerabilities. 5G networks will be vulnerable because current signaling protocols (SS7) developed for 4G networks may be critically insecure. Updating and securing these protocols will go a long way towards increasing the cyber security of 5G networks. Another threat to 5G networks is the large amount of data stored in the cloud rather than on more secure local servers. The data gathered by IoT devices contribute to a vastly expanded attack surface. Moreover, many devices have security holes, such as network backdoors. The backdoors allow malicious actors to gain a target location, eavesdrop on calls, and potentially inject ransomware into 5G networks targeted at mobile operators. Other vulnerabilities in wireless and IoT fields include SIM card vulnerabilities, Authenticated Key Exchange Protocol (AKA), and many base-station backdoor vulnerabilities [4,5,6]. Therefore, CTCs are an important alternative and crucial in the future for sending sensitive information in several confidential applications of IoT.

Covert channels can be implemented at different layers of the TCP/IP protocol stacks and can be used for the authentication of data sources, time, and content. Using protocol headers enables fundamental features, such as simultaneous connections, dynamic routing, or session management for covert channels, that enrich communications and become more adaptive and stealthy. However, a covert channel’s effectiveness is different for each layer of the TCP/IP protocol stacks. At the link layer, covert channels are limited to locally connected communication devices and cannot communicate outside the local network [7]. While at the application layer, focused on protocols with process-to-process communications over the network, covert channels are limited to the infrastructure offered by those applications. Therefore, using covert channels at the transport layer is beneficial because covert communication can use global Internet connectivity with increasing access to various types of devices and a potential expansion towards various communication areas.

This paper focuses on covert channels that transmit covert information over TCP/IP, specifically whenever the time properties of legitimate traffic are used and provides the threshold of packet delay that can accurately distinguish covert traffic from legitimate traffic, where this threshold will save the time of future researchers who are studying either the detection or mitigation of covert timing channels in various applications. Our contributions can be summarized below:

1. Analyze the behavior of covert traffic and legitimate traffic in two different networks using statistical metrics to explore how the inter-arrival times of legitimate traffic can be used effectively to transmit the covert traffic between two entities communicating over networks, and how the network conditions can affect the behavior of both traffics.
2. Find the threshold for delaying packet that makes the covert channel efficient to leak information. This threshold is really important to answer the following questions in different scenarios:
  - Scenario 1 (Covert timing channel as a cyber-attack such as leaking information between two devices)
  Does this covert timing channel really dangerous and need to be mitigated or detected?
  - Scenario 2 (Covert timing channel as confidential applications such as sending secret data in military mission)
  Does this covert timing channel reliable to send data?

The paper offers a brief overview of the literature on the design and detection of covert timing channels, discussed in Section 2. Section 3 demonstrate details the designing of experiments and discusses the transmission delay aspects adopted in this paper. Section 4 presents the accuracy of distinguishing between the binary encoding symbols and the transmission bit rate analysis is presented. Finally, the paper is concluded in Section 5.

## 2. Related Work

In this section, valuable information about covert timing channels designed and detection methods will be provided.

### 2.1. Covert Timing Channel Design Approaches

A lot of research in the literature was done in attempting to model covert timing channels. The idea of covert timing channels was formulated in 1978 based on a study by Padlipsky in [8]. Since then, it has been enriched with diverse proposals. For example, in [9], a simple synchronized covert timing channel proposed that used an on-off switching technique to embed covert binary bits in the legitimate traffic within a specific time interval. The sender transmits a packet within each particular time interval to represent bit 1 in the covert data. It does not send any packet to represent a bit 0. Then the receiver scanned the stream of traffic for incoming packets at each interval and decoded the bit as 0 or 1. The encoding method in this approach is simple, and the idea is very attractive. However, this approach requires strict synchronization between the sender and receiver, and loss the synchronization will lead to error propagation. Moreover, in [10], there was more details about the on-off covert timing channels analysis, organized them into two fields: deterministic and non-deterministic channels, and provides a method for calculating the maximum transmission bit-rate in these channels based on the packet delay distribution. In [11], a similar mechanism was proposed to encode the binary symbols where bit 0 is denoted by a fixed short time delay, and a bit 1 is denoted by a fixed delay to hide covert traffic by using a sequence of inter-packet delays.

However, other approaches were designed without time synchronization restrictions by using different timing techniques such as using the arrival pattern of packets [8,12]. Later Cabuk in [13] designed a more advanced covert timing channel mechanism which is called time-replay channel, where the inter-packet delay sequence was divided into two parts ($S_{0},S_{1}$) based on the specific threshold that is negotiated by the sender and receiver beforehand. Then the sender replays one inter-packet delay randomly from first part $S_{0}$ to send a bit 0 and replays one inter-packet delay randomly from first part $S_{1}$ to send a bit 1. This mechanism makes Time-replay covert timing channel undetectable than other channels designed before because the covert traffic is approximately close to legitimate traffic. A sophisticate covert timing channel mechanism was proposed in [14], called a model-based covert timing channel. This mechanism was designed to choose a traffic model that can fit the legitimate traffic and has the smallest root mean squared error between it and legitimate traffic. The model-based covert timing channel used Maximum Likelihood Estimation to determine the required parameters for the model, where these parameters vary to capture the variation of legitimate traffic. Using this traffic model, covert information was transformed into a sequence of random inter-packet delays.

Moreover, there are covert timing channels were designed based on different application protocols. For example, the Keyboard JitterBug algorithm was proposed in [15], for leaking covert information by adding different delays to the interval time of keystrokes to encode a bit 0 and a bit 1. This application environment was interactive communication, such as Telnet and instant messaging. The method was proposed in [16], used the Patero distribution of legitimate telnet traffic to design undetectable covert timing channel and increased the capacity of the channel using encoding “L-bits to n-packets” scheme. In [17], the authors designed a covert timing channel using the properties of inter-arrival times of legitimate network traffic and proved that these properties are valuable information for the designed detection methods to detect such channels. Moreover, in [18], a covert channel was proposed based on the postponing or extending of silence periods in voiceoverLTE (VoLTE) traffic to modulate the covert packets. The author employed Gray mode to encode the covert message and to reduce the impact of packet loss. The experiment results showed that the proposed covert channel enabled a tradeoff between the robustness and voice quality, which is an important performance indicator for VoLTE, undetectable by statistical tests, and which outperformed the other covert channels based on inter-packet delays in terms of robustness. In [19], a general overview of physical-layer network coding (PNC) in a Two-way Relay Channel (TWRC) was provided. The authors described the main encoding and decoding schemes for channel-coded PNC. Based on simulation results, the authors illustrated the decoding performance of different channel-coded PNC schemes over different types of channels. Recently, in [20], the authors proposed covert timing channels in a shared deterministic and work-conserving first-come-first serve (FCFS) scheduler.The authors presented an information-theoretic framework to describe and model the data transmission in the channels and calculate its capacity. In their model, the encoder and the decoder users used shared the packet scheduler, using indirect communication between them to send the secret message. The encoder user encoded a message in his traffic pattern, and then the decoder user can predict the message by estimating the encoders traffic pattern by the delays he experiences. Their results demonstrated the possibility of significant information leakage and greater privacy threats brought by covert timing channels in FCFS schedulers.

Some of existing techniques also used for encoding covert information into the IP Time To Live (TTL) field, and these were grouped into two classes: mapped encoding and differential encoding. In mapped encoding the covert bits were encoded by mapping the bit values to specific TTL values. For example, in [21], the original TTL value represented a bit 0, and the TTL value was increased by an integer, Δ, to represent a bit 1. While differential encoding, covert bits were encoded by the changes between subsequent TTL values. For example, in [22], a bit 1 was encoded when TTL increased by Δ, and a bit 0 was encoded when TTL decreased by Δ. In [23], the original TTL value represented a bit 0 and a TTL value decreased by Δ represented a bit 1. Therefore, the transmission times for covert timing channel packets increased or decreased based on adding or subtracting from the TTL value when the covert information was encoded into the TTL field.

Most of the researchers who used the time properties of legitimate traffic to design undetectable covert timing channels used a single network environment for that and ignored analysis of the behavior of their methods on other networks. This paper focus on using the time properties of legitimate traffic to send covert data in two different network environments to explore the behaviour of covert timing channel in both of networks and examine how network conditions affect on detection such channels.

### 2.2. Covert Timing Channels Detection Approaches

Covert timing channel detection methods mainly used the statistical tests to differentiate covert traffic from legitimate traffic by examined the shape and regularity of inter-arrival times and inter-packet delays. The statistical tests can be categorized mainly into three classes: The shape tests, regularity tests, and entropy tests. For example, in [24] the authors proposed Kolomogorov-Smirnov (K-S) shape test for detecting covert timing channels. The K-S test was used to quantify the distance between two empirical distributions of covert traffic and legitimate traffic. When the distance between the two distribution was large, this indicates the presence of covert timing channels in the legitimate traffic. The regularity test was proposed in [9], using the standard deviation metric to detect the existence of covert timing channels. In this test the high values of standard deviation provided a strong indication of existence of covert timing channels and low values imply the absence of such channels. Moreover, the entropy test used in [25,26] as measure to detect covert timing channels based on the knowledge that the covert timing channel mechanism must cause some effects on entropy of legitimate traffic. Entropy can provide significant evidence for the existence of pattern inside the data singling the presence of a covert timing channel. High entropy indicates a high randomness in the data and absence of a covert timing channel, whereas low entropy indicates a high consistency in data and high possibility of covert channels existence.

In order to overcome the issues in detecting covert timing channels based on flat statistical-based analysis (e.g., calculating statistical tests at one-scale level), a hierarchical statistical method was proposed in [27] to determine the existence of covert timing channels in a flow of inter-arrival times. Their results showed a significant capability for detecting such channels using the hierarchical based-statistical method when compared to the flat statistical-based method. As an extension of the work originally conducted in [27], the authors in [28] used the MapReduce technique to improve the speed of covert timing channel detection using the hierarchical statistical-based method. Their method presented good accurate results in detecting such channels within a reasonable time compared to the sequential hierarchical method.

Machine learning also used as another method to detect covert timing channels. The Support Vector Machine (SVM) classifier was widely used in the covert-channel detection, such as in [29] the authors used SVM to detect four different covert timing channel algorithms. Decision Tree classifier also used in [30] for identifying and detecting covert timing channels. Moreover, in order to improve the accuracy of covert channel detection, the authors in [31,32], used unsupervised machine learning methods to classify and detect covert timing channels based on the statistical properties of network traffic. Recently, in [33], a new covert timing channel detection method proposed based on using deep neural networks and a set of statistical features extracted from the flow of inter-arrival times using the hierarchical statistical-based method. The study results showed that deep neural networks achieved a better level of accuracy and significantly shorter training model time compared with SVM.

## 3. Methodology

This section describes design of the experiments that conducted using real network traffic to transmit covert information using covert timing channels between the sender and receiver in two different network configurations.

### 3.1. Experimental Design

The scope of this study focuses on using the time of legitimate traffic to transmit covert information between the sender and receiver over the network bypassing anomaly detection methods. Based on the study’s goal, a covert timing channel application was designed and run using reliable TCP/IP networks. The application was implemented in Java and consisted of a server program and a client program, which represent the sender and the receiver, respectively. In the discussion that follows, we use particular terminology while talking about covert timing channels. The *sender* of the covert timing channel is responsible for modulating the time transmission of legitimate traffic to transmit covert information using socket API, and to which the receiver has access. The *receiver* observes the covert traffic, extracts the timing information, and decodes the covert bits. For a better understanding of the application method, it is important to clarify different terms related to time properties that be used. ($T_{s}$), is the actual sending time of the packet that transmitted to the receiver. ($T_{r}$), is the actual receiving time of the packet and IAT is the time interval between the receiving time of two packets on the receiver side. In addition, the actual transmission time of packet is added by additional time (ϵ) due to the network conditions, such as queuing time between subsequent packets. Therefore, we can define the time for sending the packets between the sender and the receiver, and ϵ, which accounts for possible variations in the network conditions, as follows:

(1) $$T_{r}=T_{S}+\epsilon$$

**Figure 1:**
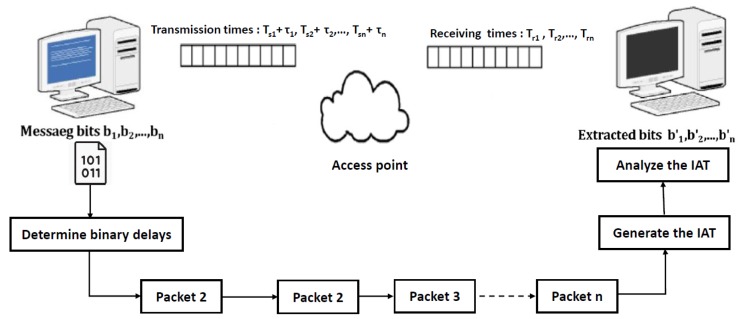
Scheme of encoding binary symbols to the inter arrival time.

The binary covert timing channels are designed as a one-way channel, where the sender does not receive any feedback from the receiver regarding the time the packet was received or whether it was correctly decoded. This limits the performance of the covert timing channel, but helps to increase the difficulty in detecting them, as shown in Figure 1.

#### Experimental Configuration Setup

To study the behaviour of covert traffic and legitimate traffic in different network conditions, the experiments of this study were run in two different network configurations (private and public), and data was transmitted between two personal computers in both network configurations. Table 1 and Table 2 describe the computer’s properties that were used in all the experiments and the network configuration properties were considered in the study, respectively.

### 3.2. Covert Timing Channel Sender

In the experiments of this study, the sender and the receiver did not require synchronized clocks between them but did need (individual) clocks with sufficient accuracy. Therefore, the PC devices’ crystal-controlled clock was used to govern timing. It is necessary to understand the process of encoding covert information and sending this data between the sender and the receiver from both sides. First, the sender generates a secret message, then converts the messages’ characters to binary format and saves it as a text file. Then, the binary covert packets will inject in the legitimate packets by modulating the transmission times of these packets ${T_{s}}_{i}$ by adding extra buffering delays denoted by ($\tau_{i}$), to each element of the original covert sequence of information, as shown in Equation (2).

(2) $${{T^{\prime }}_{s}}_{i}={T_{s}}_{i}+\tau_{i}+\epsilon$$

For the binary encoding symbols the delays were defined based on a form of Morse code. A short delay was added to the $T_{s}$ to encode a bit 0, and a long delay was added to the $T_{s}$ to encode a bit 1. A delay value of zero indicated that no extra delay was added, and no cover information was sent out. In other words, the resulting sequence of *n*-bit binary symbols were represented in a sequence of *n* packet transmission time $\left\{{{T^{\prime }}_{s}}_{1},{{T^{\prime }}_{s}}_{2},\ldots ,{{T^{\prime }}_{s}}_{n}\right\}$ in the differences, such that:

$${{T^{\prime }}_{s}}_{i}=\left\{\begin{array}{cc} {T_{s}}_{i}+{\tau_{b}}_{i}+\epsilon & \mathrm{if}\;\;b_{i}=0\\ {T_{s}}_{i}+{\tau_{b}}_{i}+\epsilon & \mathrm{if}\;\;b_{i}=1 \end{array}\right.$$

In the previous studies, the binary encoding symbols were used with delays to inject the binary covert packets within the legitimate traffic between the entities communicating over the networks based on assuming a fixed time window. However, this study examined using the binary encoding symbols with different delays in two network configurations to inject the binary covert packets within the legitimate traffic without using fixed time windows. In our experiments, the mean inter-arrival times of legitimate traffic were used to determine the buffering delays that can be used to inject the covert traffic in the legitimate traffic. Based on the experiments, the mean (μ) was found equals ≈0.0050 s and ≈0.0025 s in the network configurations 1 and 2, respectively. Then the time range of legitimate traffic was measured using the *2 Sigma* control limits, which consists the upper control limit ($L_{u}$) was measured as 2 sigma above the μ, and lower control limit ($L_{l}$) was measured as 2 sigma below the μ, same as shown in Equations (3) and (4). The time range of legitimate traffic was found within two time ranges, $\left[0.003109,0.007274\right]$ seconds and $\left[0.001254,0.004311\right]$ seconds in network configurations 1 and 2, respectively.

(3) $$L_{u}=\mu +2\times \sigma \;$$

(4) $$L_{l}=\mu -2\times \sigma \;$$

To determine the threshold of packet delay that can accurately distinguish covert traffic from legitimate traffic, five cases were examined in two different network environments. Each case represents a certain value (λ) that can be used to determine the delays whether the covert packets 0 or 1 based on the mean inter-arrival times of legitimate packets, where the short delay of a binary bit 0 equals λ, and a long delay for a binary bit 1 equals (2×λ), and λ value defined in the equation below:

(5) λ=c×μwherec={0.025,0.50,1.00,2.00,3.00}

For example, in the case of (λ=0.025×μ) in the network configuration 1 when μ is 0.0050 seconds, the delays are 0.001250 second and 0.002500 for binary covert packets 0 and 1, respectively. The following table shows the delays used for the binary covert packets in both network configurations.

### 3.3. Covert Timing Channel Receiver

For the covert timing channel, the receiver is a passive eavesdropper that needs to measure the times at which each network packet arrived. In covert timing channels, two types of times can be used in the receiver side to extract the timing information of packets: TCP Timestamps and sniffer timestamps. TCP timestamps are corresponding to the times at which the network packets are sent according to the source clock, which are unaffected by network conditions. The main disadvantage of TCP timestamps is it coarser granularity on many operating systems and demanding the use of large timing windows for symbol encoding and decoding. While sniffer timestamps correspond to the times the time at which packets are seen by a remote network sniffer. Due to network delays, these timestamps are offset from the actual time the packet was sent at the source. In addition, these timing offsets are affected by any network conditions. Therefore, sniffer timestamps are more conservative assumptions for anyone who wants to use these types of times to send data using covert timing channels.

**Figure 2:**
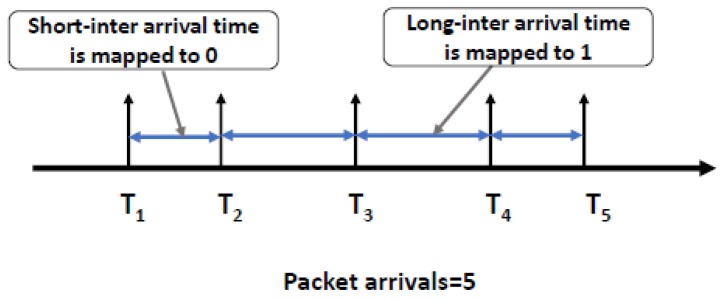
Representation of binary symbols in the packet inter-arrival times.

On the receiver’s side, the receiver records the sequence of receiving times $\left\{{T_{r}}_{i}\right\}$ of network packets corresponding to each binary covert packets. Then, the sequence of inter-arrival times of $\left\{IAT_{1},IAT_{2},\ldots ,IAT_{n}\right\}$ maps to the *n*-bit binary sequence same as shown in Figure 2, where $\left\{IAT\;=\;{T_{r}}_{i}-{T_{r}}_{i-1}\right\}$. In the last step, the binary symbols re-organized to reassemble the original message.

In this study, the total flow size of 51,200 inter-arrival times were collected, wherein each experiment, a binary stream with size 1024-bits was injected within the legitimate traffic in both network configurations considering the different value of λ. In practice, the monitor program Wireshark was used to track and collect the receiving times of the packets on the receiver side, based on the five-tuple definitions often used in network traffic analysis, consisting of:

1. Source IP address
2. Destination IP address
3. Source port
4. Destination port
5. Protocol

Because the receiver uses inter-arrival times of packets and not absolute packet times, there is no need for synchronization between the sender and receiver clocks. The sender sends the binary covert packets using the delays that represent in Table 3 in both network configurations, then the receiver measured the inter-arrival times of these packets. The upper and lower control limits of inter-arrival times of binary covert packets in each experiment of each λ value were calculated using Equations (3) and (4). Then the time range of binary covert packets in each case defined using the mean of upper and lower control limits for all experiments were used in that case, as shown in the following equations:

(6) $$l_{u}=\frac{\sum_{n=1}^{N}{l_{u}}_{n}}{N}$$

(7) $$l_{l}=\frac{\sum_{n=1}^{N}{l_{l}}_{n}}{N}$$

where *N*, is the number of experiments. By calculating the mean value for the upper and lower control limits for all experiments, the time ranges for the binary covert packets were recorded in Table 4 and Table 5 in both network configurations. The left-hand columns give the delays for binary covert packets and the remaining two columns give the time range of inter-arrival times of these packets and the probability the packets were received in that range based on a given delay, respectively. These particular tables were obtained from experiments that were running during a workday in August 2019 in both of network configurations. For example, the first row in Table 4 represents binary covert packets sent using 0.001250 s and 0.002500 s delays, and 97% of the zero-bit packets were received within range $\left[0.003475,0.009128\right]$ s, and 96% of one-bit packets were received within range $\left[0.007044,0.009528\right]$ s. These results are similar to the rows in both Table 4 and Table 5.

There are some difficulties in this approach. For example, the entries in the previous Table 4 and Table 5 were not constant over time, they changed depending on the network conditions, such as the delay of packets caused by network congestions or other dynamic conditions. Therefore, patterns of network traffic can be kept dynamic in real time, and hackers will face the same problem of dynamic network conditions when they try to choose secret exfiltration parameters to use the network environment to leak the highest amount of data.

## 4. Accuracy and Transmission Bit Rate Analysis

This section describes the accuracy of distinguishing covert traffic from legitimate traffic in two different network configurations based on using different values of λ, and the transmission bit rate of covert timing channels in two network configurations using different values of λ, and different percentage of binary packets.

### 4.1. Accuracy of Distinguishing Covert Traffic from Legitimate Traffic

This section illustrates how the accuracy of distinguishing covert traffic from legitimate traffic was measured and how it is possible to detect covert traffic by observing the time range of this traffic. The accuracy is measured based on the percentage of correctly distinguish inter-arrival times of covert traffic from inter-arrival times of legitimate traffic based on the time range for both traffics. Where finding thresholds that can accurately distinguish between covert and legitimate traffic is important in different applications, such as applications are designed to make the covert timing channels undetectable for malicious activities or secure for transmitting sensitive information.

**Figure 3:**
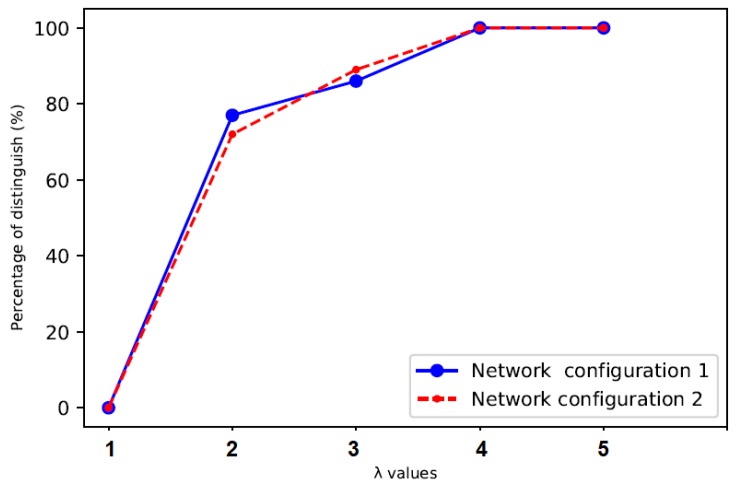
Accuracy of distinguishing covert traffic from legitimate traffic.

The experiment results in Figure 3 show the accuracy is 0% in both network configurations when λ is a quarter the mean inter-arrival time of legitimate traffic. More covert traffic are counted close to the legitimate traffic, making the time range of covert traffic overlaps with the time range of legitimate traffic and the distinction between them hard. However, when λ value increased, such as in cases 2 and 3, the percentage of distinguishing between covert traffic and legitimate traffic increased as well, which becomes possible to distinguish between them with a significant degree between 70–80%. The accuracy reached 100% when λ is a double the mean inter-arrival times of legitimate traffic in both network configurations. Therefore, using the threshold of packet delay that are less than the double mean inter-arrival times of legitimate traffic, makes a small difference between the time ranges of covert traffic and time ranges of legitimate traffic. Furthermore, in both network configurations, at any time after the double mean inter-arrival times of legitimate traffic, the accuracy will remain at 100%. This indicates that it is easy to distinguish covert traffic from legitimate traffic. Based on that, covert timing channels will be *dangerous* in the case of malicious applications or *secure* for confidential applications.

### 4.2. Transmission Bit Rate

**Figure 4:**
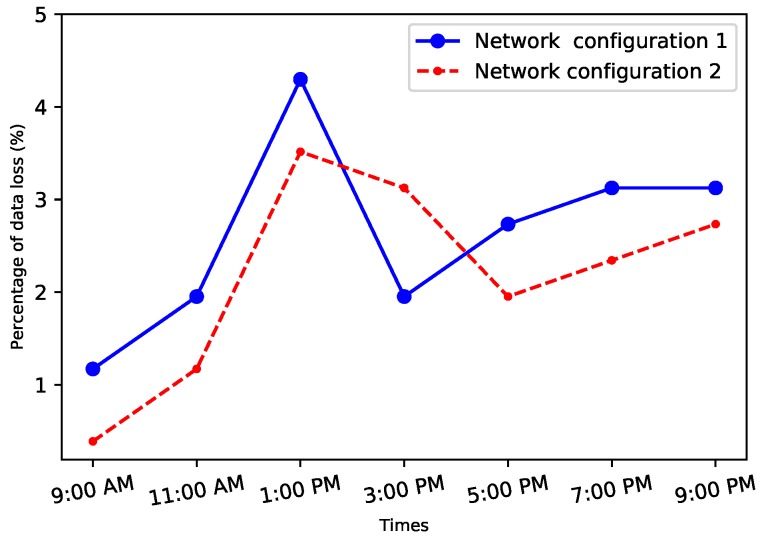
Percentage of data loss at different time windows.

In this study, the sender and the receiver were assumed to have gained control over one of the systems’ resources. They started by conducting an investigation of the network characteristics around the system, with the intent of discovering the optimal parameters of the network environment. For example, finding the time when the network uncongested to use this time for leaking the highest amount of information, such as Figure 4 shows the loss of data at different time windows to explore the congestion issues in different environments. The results show the highest losing of data was at rush hours between 11:00 a.m. and 1:00 p.m.

Although the loss of packets was the highest at that time but still the percentage of losing the packets was very low and does not exceed 5%.

The covert timing channel that was used in this study is not symmetric, where the transmission delays are different for the binary covert packets and transmission bit rates are different as well. In other words, it takes less time to transmit 1024-bits of zeros than to transmit 1024-bits of ones. This indicates that the transmitted bit rate of zeros is higher than the transmitted bit rate at which ones are transmitted.

**Figure 5:**
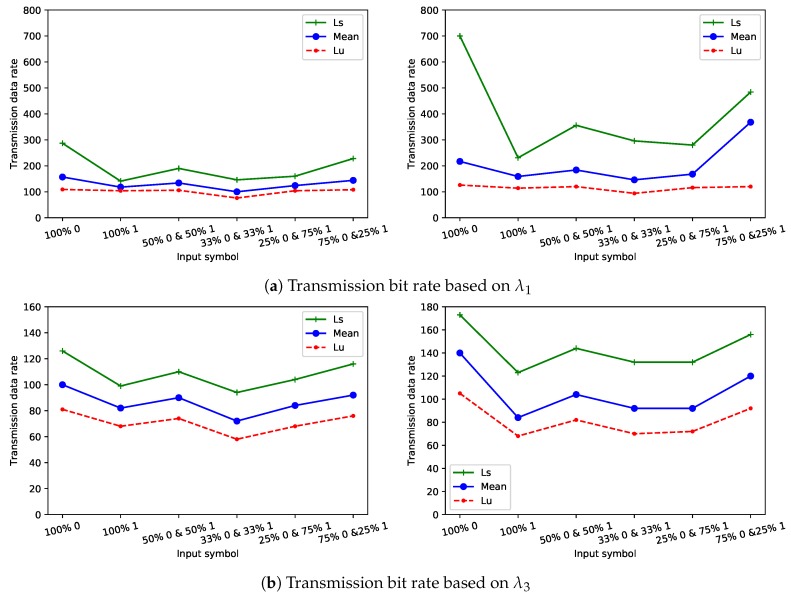

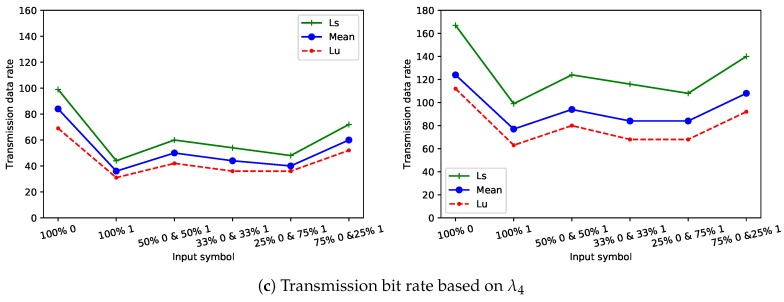
Transmitted bit rates of network configuration 1 (left) and network configuration 2 (right).

The fact that zeros and ones are intermixed in the communication experiments does matter, as the results would have been different if the percentage of zeros and ones in the covert information was different. To analyze the impact of intermixed zeros and ones within covert information on transmitted bit rate, different scenarios were examined, where each scenario introduced different percentages of zeros and ones in the covert information. One of the scenarios that used was 50% zeros and 50% indicating that when the binary encoding stream with a length equals to 1024-bits were transmitted between the sender and the receiver, the stream contained 512 bit of zeros and 512 bits of ones. This was similar for all other scenarios, and each scenario was examined using three metrics: $L_{l}$, mean, and $L_{u}$ for inter-arrival times ranges of binary covert packets, which are represented in Figure 5. The results showed that the highest number of bits that can be transmitted through the covert timing channel was recorded when short delays were used in both network configurations, as shown in Figure 5a. As the difference between delays increased, the transmission bit rates decreased, as shown in Figure 5b,c in both network configurations. Moreover, the number of bits that were sent, based on the $L_{u}$ value in the range of the inter-arrival times, was low when compared to the number of bits that were sent based on the $L_{l}$ and mean for any transmission delays in both network configurations. This is because 1 s in configuration 1 is considered insufficient time to send a large number of bits with high delays and sending a mount of bits depends on the transmission speed of the network. For calculating the bit transmission rate results that are shown in Figure 5 the following formula was used:

(8) $${T_{d}}_{\left(4-bits\right)}=N_{0}\times V_{0}+N_{1}\times V_{1}$$

where ${T_{d}}_{\left(4-bits\right)}$ is the time duration needed to send a binary data stream with length is 4 bits; $N_{0}$ is the number of zeros in the 4-bits stream; $V_{0}$ is one of the three values of $L_{l}$, mean, or $L_{u}$ for bit 0, $N_{1}$ is the number of ones in the 4-bits stream; and $V_{1}$$V_{0}$ is one of the three values of $L_{l}$, mean, or $L_{u}$ for bit 1. In Equation (8), a data stream with a size is 4 bits was chosen for simplicity to calculate the transmission bit rate based on the percentage of zeros and ones in the covert information. For example, in the scenario that introduces 75% zeros and 25% ones at delay $\tau_{0}=0.00125$ s and delay $\tau_{1}=0.0025$ s in network configuration 1, where the minimum values ($L_{l}$) in the range of inter-arrival times for zeros and ones are 0.003475 and 0.007044, respectively, the 4-bits stream contained 3 bits of zeros and 1 bit of ones, and the time duration to send these 4 bits was equal to (3×0.003475)+(1×0.007044)=0.017469 s. Then, the number of streams that needed to transmit the 4 intermixed binary encoded symbols based on the time duration of the bits determined in Equation (8) was defined in the following formula:

(9) $$N_{T_{d}}=\left\lfloor \frac{T_{u}}{{T_{d}}_{\left(4-bits\right)}}\right\rfloor$$

where $T_{u}$ is the time unit used when the covert information is sent between the sender and receiver. In this study, the time unit is 1 second. Then, the number of binary bits that were transmitted in per second in all 4-bit streams was calculated based on the formulas below:

(10) $$TR_{0}=N_{0}\times N_{\left(T_{d}\right)}$$

(11) $$TR_{1}=N_{1}\times N_{\left(T_{d}\right)}$$

## 5. Conclusions and Future Works

This study used covert timing channels by modifying the time of legitimate traffic and inject the traffic that contains covert information within the legitimate traffic. The inter-arrival times of legitimate traffic and covert traffic were analyzed in two different network configurations to explore the behaviour for both traffics in both configurations and observe how the network conditions affected on the bite rate transmission of the covert timing channels and the accuracy of distinguishing covert traffic from legitimate traffic.

Our results found that the covert traffic did not usually exhibit extreme values when the threshold of packet delays that used to hide the covert data less than or equal the quarter of the mean of the inter-arrival times of legitimate traffic. Therefore, more covert traffic are counted close to the legitimate traffic, making the time range of covert traffic overlaps with the time range of legitimate traffic and the distinction between them hard. However, there is no overlap between the time ranges of covert traffic and the time range of legitimate traffic when the threshold of packet delays that used approximately equal to or greater than the double the mean inter-arrival times of legitimate traffic, making the distinguishing between them easier. Based on these observations, it is useful to find these threshold that can help to distinguish between the covert from legitimate traffic. This threshold can be used to design undetectable TCP/ IP covert timing channels that can be used for different purposes.

In this paper, we deliberately avoided optimizing for any particular channel or networked application, instead of identifying parameters that give the satisfactory performance and that remain highly robust under varied conditions. Therefore, one of extensions to this work is development of better framing and encoding schemes that are robust and general enough to work under any unknown environment without affecting user perception, by making less conservative assumptions that take advantage of specific channel or network properties. For this extension, more problem domain and statistical related features will be added. For the domain related features, we will consider two broad classes of features: hardware related, such as network speed, network size, and number of hops; and software related, such as type of applications, type of networks, type of protocols such as UDP, and type of error-correction algorithms, such as low-density parity-check (LDPC) codes [34,35] for transmitting a covert message with high reliability over a noisy transmission channel. For the statistical features, we will consider other metrics that include mode, auto-correlation, and inter-arrival time distribution. Moreover, for the other extensions to this work are using machine learning algorithms to predict the patterns the network traffic based on multiple parameters of the networks, and compare the proposed method with other previous methods in different network configurations to demonstrate the ability to distinguish covert traffic from legitimate traffic based on the threshold of packet delays.

## Figures and Tables

**Table 1:** Devices properties.

	PC 1 (Sender)	PC 2 (Receiver)
Processor	Intel(R) Core(TM) i5-4210U	Intel(R) Core(TM) i7-6500U
CPU speed	1.70 GHz 2.40 GHz	2.50 GHz 2.60 GHz
RAM	6.00 GB	8.00 GB
System type	64-bits	64-bits
Adapter type	Ethernet 802.3	Ethernet 802.3

**Table 2:** Network configuration characteristics.

	Network Configuration 1 (Private)	Network Configuration 2 (Public)
Internet speed	52.1 mbps download 15.9 mbps upload	42.8 mbps download 47.1 mbps upload
Latency	55 ms	58 ms
Router type	Home hub 3000	D-link
Number of hops	1	1
Geographical location	Personal use network St John’s, NL	Research lab at Memorial University John’s, NL

**Table 3:** Binary covert packet delays.

	Network Configuration1			Network Configuration 2	
λ	*c* Value	Zero Delays	One Delays	Zero Delays	Zero Delays
$\lambda_{1}$	0.025	0.001250	0.002500	0.000625	0.001250
$\lambda_{2}$	0.500	0.002500	0.005000	0.001250	0.002500
$\lambda_{3}$	10.00	0.005000	0.010000	0.002500	0.005000
$\lambda_{4}$	20.00	0.010000	0.020000	0.005000	0.010000
$\lambda_{5}$	30.00	0.015000	0.030000	0.007500	0.015000

**Table 4:** Time ranges of binary covert packets in network configuration 1.

λ	Binary Symbol	Binary Delays (Seconds)	Time Range (Seconds)	Probabilities
$\lambda_{1}$	Zero	0.001250	$\left[0.003475,0.009128\right]$	0.97
	One	0.002500	$\left[0.007044,0.009528\right]$	0.96
$\lambda_{2}$	Zero	0.002500	$\left[0.007241,0.009269\right]$	0.98
	One	0.005000	$\left[0.007437,0.012877\right]$	0.97
$\lambda_{3}$	Zero	0.005000	$\left[0.007580,0.012301\right]$	0.97
	One	0.010000	$\left[0.010052,0.014773\right]$	0.98
$\lambda_{4}$	Zero	0.010000	$\left[0.010077,0.014489\right]$	0.98
	One	0.020000	$\left[0.022679,0.031679\right]$	0.97
$\lambda_{5}$	Zero	0.015000	$\left[0.017722,0.023021\right]$	0.98
	One	0.030000	$\left[0.032630,0.036662\right]$	0.98

**Table 5:** Time ranges of binary covert traffic in network configuration 2.

λ	Binary Symbol	Binary Delays (Seconds)	Time Range (Seconds)	Probabilities
$\lambda_{1}$	Zero	0.000625	$\left[0.001754,0.006125\right]$	0.96
	One	0.001250	$\left[0.001685,0.007821\right]$	0.95
$\lambda_{2}$	Zero	0.001250	$\left[0.001795,0.007915\right]$	0.96
	One	0.002500	$\left[0.004315,0.008203\right]$	0.95
$\lambda_{3}$	Zero	0.002500	$\left[0.004515,0.008777\right]$	0.96
	One	0.005000	$\left[0.004995,0.009020\right]$	0.96
$\lambda_{4}$	Zero	0.005000	$\left[0.005985,0.008864\right]$	0.96
	One	0.010000	$\left[0.010029,0.015721\right]$	0.97
$\lambda_{5}$	Zero	0.007500	$\left[0.008437,0.014877\right]$	0.97
	One	0.015000	$\left[0.015863,0.025222\right]$	0.97
